# Second primary malignancies induced by radioactive iodine treatment of differentiated thyroid carcinoma — a critical review and evaluation of the existing evidence

**DOI:** 10.1007/s00259-022-05762-4

**Published:** 2022-03-23

**Authors:** Maximilian J. Reinecke, Gerrit Ahlers, Andreas Burchert, Friederike Eilsberger, Glenn D. Flux, Robert J. Marlowe, Hans-Helge Mueller, Christoph Reiners, Fenja Rohde, Hanneke M. van Santen, Markus Luster

**Affiliations:** 1 Department of Nuclear Medicine, University Hospital Marburg, Marburg, Germany; 2 Department of Internal Medicine, Hematology, Oncology and Immunology, University Hospital Marburg, Marburg, Germany; 3 Department of Physics, Royal Marsden Hospital and Institute of Cancer Research, Sutton, UK; 4 Spencer-Fontayne Corporation, Jersey City, NJ 07304-1901 USA; 5 Institute for Medical Bioinformatics and Biostatistics, Philipps University of Marburg, Marburg, Germany; 6 Department of Nuclear Medicine, University Hospital Wuerzburg, Wuerzburg, Germany; 7 Department of Pediatrics, Wilhelmina Children’s Hospital, University Medical Center Utrecht, Utrecht, The Netherlands; 8 Princess Máxima Center for Pediatric Oncology, Utrecht, The Netherlands

**Keywords:** Differentiated thyroid carcinoma, Radioiodine therapy, Second primary malignancy, Hematologic malignancy, Dose–response relationship, Effect of dose on second primary malignancy risk

## Abstract

**Purpose:**

Concern is growing about long-term side effects of differentiated thyroid cancer treatment, most notably radioactive iodine (RAI) therapy. However, published studies on the subject have had heterogeneous cohorts and conflicting results. This review seeks to provide an updated evaluation of published evidence, and to elucidate the risk of second primary malignancies (SPMs), especially secondary hematologic malignancies (SHMs), attributable to RAI therapy.

**Methods:**

An extensive literature search was performed in Ovid MEDLINE, Ovid MEDLINE and In-Process & Other Non-Indexed Citations, Ovid MEDLINE Epub Ahead of Print, Cochrane Central Register of Controlled Trials (CENTRAL) and PubMed. Studies regarding RAI-induced SPMs or a dose–response relationship between RAI therapy and SPMs were identified, 10 of which were eligible for the analysis. We evaluated risk of bias in each study and judged quality of evidence (QOE) across all studies using the Grading of Recommendations, Assessment, Development and Evaluations approach.

**Results:**

For the outcome “SPM”, the relative effect (relative risk, hazard ratio, or odds ratio) of RAI vs. no RAI ranged from 1.14 to 1.84 across studies, but most results were not statistically significant. For the outcome “SHM”, reported relative effects ranged from 1.30 to 2.50, with 2/3 of the studies presenting statistically significant results. In 7/8 of the studies, increased risk for SPM was shown with increasing cumulative RAI activity. QOE was “very low” regarding SPM after RAI and regarding a dose–response relationship, and “low” for SHM after RAI.

**Conclusion:**

Based on low quality evidence, an excess risk for the development of SPM cannot be excluded but is expected to be small.

**Supplementary Information:**

The online version contains supplementary material available at 10.1007/s00259-022-05762-4.

## Introduction

Thyroid cancer, estimated to be the sixth most common carcinoma in the USA in 2021 in women, is a malignancy with increasing incidence in the last decades [1]. This trend has been driven by an increased incidence of differentiated thyroid cancer (DTC), most notably small papillary thyroid carcinoma [2–5]. As these histological types of thyroid cancer have a generally favorable prognosis with 10-year survival above 90%, concern is growing about late adverse effects of DTC treatment that might impair survivors’ quality-of-life, or even themselves prove life-threatening [6, 7].

After surgery, radioactive iodine (RAI) for remnant ablation or adjuvant therapy is recommended for high-risk DTC and, notwithstanding debate among experts [8, 9], is also recommended for substantial proportions of intermediate-risk and low-risk DTC cases, depending on national guidelines [10–12]. Side effects of RAI treatment may include nausea and vomiting, radiation thyroiditis, and in rare cases, sialadenitis and xerostomia, bone marrow suppression, gonadal dysfunction, second primary malignancies (SPMs), and in the presence of widespread lung disease, pulmonary fibrosis [13, 14]. As long-term adverse effects are a substantial component of patient-relevant outcomes, any risk–benefit ratio has to incorporate the potential for SPM occurrence. The relationship of RAI and SPM, if any, is the subject of ongoing discussion, and existing evidence is conflicting. While some authors found an increased risk of SPM related to RAI therapy [15–18], others could not reproduce these findings, or even observed a lower risk of SPM in the exposed group compared with patients not undergoing adjuvant [^131^I]NaI therapy [19–22]. Prior reviews and meta-analyses showed enormous heterogeneity between studies [23, 24]. We sought to perform an updated critical review of available data concerning the risk of SPM in patients with DTC undergoing post-operative RAI therapy, compared to the risk in their counterparts undergoing surgery alone. As part of this review, we sought to elucidate the quality of the published evidence. Outcomes of interest were: (1) occurrence of any SPM or (2) any secondary hematologic malignancy (SHM), and (3) evidence of a dose–response relationship between the cumulative administered activity of RAI and the risk of SPM. Throughout this paper, we use the term “dose–response relationship” recognizing that it may be more accurate to refer to an “association” between administered ^131^I activity and occurrence of SPM, than to a “relationship”, since the latter term may imply a deterministic rather than a stochastic effect.

## Methods

**Fig. 1:**
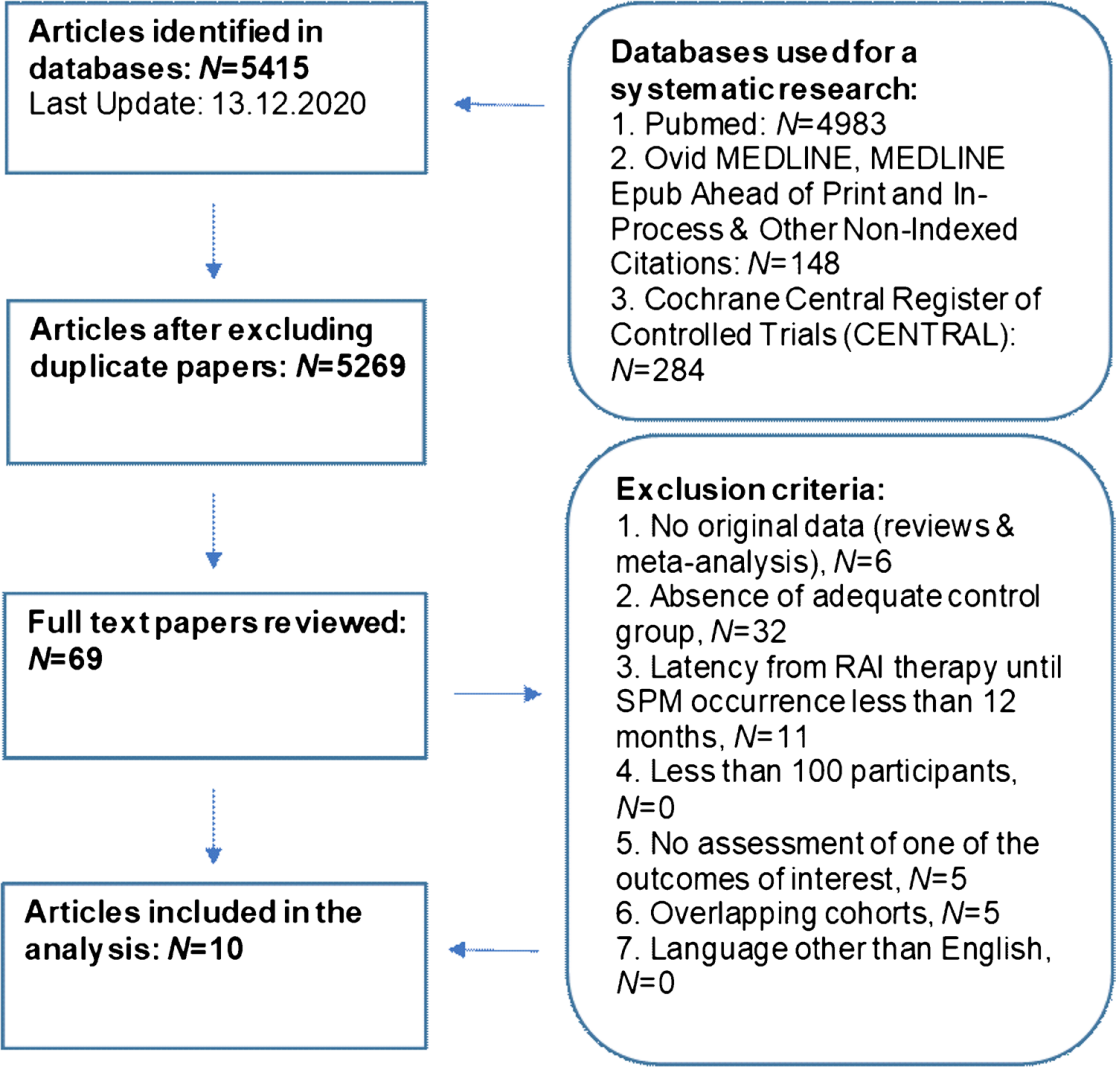
Research process and inclusion of studies. Regarding the exclusion criteria, every paper was counted only once, since the exclusion criteria were applied stepwise. Abbreviations: RAI, radioactive iodine; SPM, second primary malignancy

We prepared this paper in accordance with the PRISMA 2020 statement for reporting systematic reviews. [25] We conducted an electronic literature search for studies examining the risk of SPM in thyroid cancer survivors treated with RAI therapy compared to survivors treated with surgery alone using the following databases: Ovid MEDLINE, Ovid MEDLINE and In-Process & Other Non-Indexed Citations, Ovid MEDLINE Epub Ahead of Print, Cochrane Central Register of Controlled Trials (CENTRAL) and PubMed. We performed an initial search on May 3^rd^, 2020 and updated the results on Dec 10^th^, 2020. The queries were “thyroid cancer” combined with “second primary cancer” and their synonyms (Online Resource 1). One author (M.R.) reviewed all citations (*N* = 5269) for relevance and abstracts were analyzed whenever suitable. Finally, 69 full-text articles were retrieved and assessed by two authors (M.R., M.L.) independently. The following exclusion criteria were applied: (1) reviews or meta-analyses, (2) absence of adequate control group, (3) < 12-month latency between DTC diagnosis or treatment and SPM occurrence, (4) < 100 participants, (5) no outcome of interest assessed, (6) overlapping cohorts, and (7) language other than English (Fig. 1).

A control group was considered appropriate if it comprised patients treated without RAI. Whenever cohorts overlapped between studies, we included the article with the most up-to-date or most extensive data. Insufficient latency between DTC and SPM was an exclusion criterion because studies suggest that radiation-induced malignancies take several years or even decades to develop [26–28]. It is noteworthy that solid cancers are expected to occur after 5 years or more, while for SHMs, a peak of excess cases within the first 5 years after radiation exposure was demonstrated [26, 29]. The paper of Rubino et al. [16] was not excluded despite featuring a meta-analysis, because the article also reported updated data regarding three included cohorts, therefore providing a larger, newer dataset than in corresponding original reports [30–32].

Three authors (M.R., G.A., M.L.) independently assessed the risk of bias (ROB) in each study with the Risk of Bias in Non-randomized Studies – of Interventions (ROBINS-I) tool and discussed results until achieving consensus. In the same manner, these authors estimated the quality of evidence (QOE) regarding each outcome using the Grading of Recommendations, Assessment, Development and Evaluations (GRADE) approach [33, 34]. Outcomes of interest were (1) occurrence of SPM, (2) occurrence of SHM, and (3) dose–response relationship.

As its name implies, the ROBINS-I tool is recommended by the Cochrane Collaboration in order to assess ROB in non-randomized studies of interventions [33]. Evaluation using ROBINS-I starts by defining a target trial, which is a desirable hypothetical randomized trial unrestricted by ethical limitations or other factors. Any potential ROB is assumed to originate from the difference between the target trial and the analyzed study in seven domains: bias (1) due to confounding, (2) in selection of participants into the study, (3) in classification of interventions, (4) due to deviations from intended interventions, (5) due to missing data, (6) in measurement of outcomes, and (7) in selection of reported results. Each domain can be classified as having either: a *low* ROB (the study was comparable to a randomized trial regarding the domain), a *moderate* ROB (the study was sound for a non-randomized study regarding this domain, but cannot be considered comparable to a well-performed randomized trial), a *serious* ROB (the study has some significant problems in this domain), a *critical* ROB (the study is too problematic in this domain to provide any useful evidence on the effects of intervention) or *no information* to judge ROB for this domain [33]. An overall ROB classification is obtained by summing the weighted results of the domain classifications. However, the ROBINS-I tool mandates that the overall ROB classification cannot be better than that of the worst domain. In contrast, several domain classifications can sum up to a worse overall judgment. For instance, a serious ROB in two or more domains can lead to the judgment of a critical overall ROB. Where different data were available for a cohort, we analyzed the data that we expected to have the lowest ROB (Online Resource 2).

The GRADE approach [34] facilitates the process of rating the quality of the best available evidence, and therefore comprises information from all studies included in a critical or systematic review or meta-analysis. The QOE is rated as *high*, *moderate*, *low*, or *very low*. A priori, the QOE provided by randomized trials is assumed to be high, whereas the QOE provided by observational studies is assumed to be low. Proceeding from these assumptions, overall QOE is determined by factors that cause an increase or decrease. Factors increasing the QOE are (1) a large magnitude of effect (i.e., relative risk [RR] > 2.0 or < 0.5), (2) all plausible confounding would reduce the demonstrated effect or increase the effect, if no effect was observed, and (3) a dose–response gradient. Factors reducing the QOE are (1) limitations in study design or execution (ROB – assessed by the ROBINS-I tool), (2) inconsistency of results, (3) indirectness of evidence, (4) imprecision, and (5) publication bias.

Our primary endpoint was relative effect, defined as any of RR, hazard ratio (HR), or odds ratio (OR), depending on the particular study’s endpoints. The extracted data were calculated and analyzed with different methods. Nevertheless, RRs and HRs can be interpreted as being similar [35]. Additionally, ORs can be interpreted in the same way as RRs, when the incidence of the observed outcomes (e.g., SPM, SHM) is rather small. A crude incidence of 5–20% is considered sufficiently low to interpret an OR as approximating an RR [36, 37]. The crude incidence rates of SPM in all included studies ranged from 1.1 to 12.0% [22, 38]. The crude incidence rates of the included studies using OR ranged from 1.1 to 2.5% [20, 38]. Therefore, we assumed that these ORs could be interpreted as RRs. A p-value < 0.05 was considered to be statistically significant.

Where possible, we used forest-plots to visualize the relative effect results of each study. Nonetheless, data from some included studies could not be presented in this manner, because the data were not comparable with the relative effects of the other studies. That was the case whenever the papers provided only data for an indirect comparison of irradiated versus non-irradiated patients, or whenever data were provided as relative effect per administered RAI activity [18, 21, 22, 39]. Excluding these studies would establish an unacceptable loss of information and might cause bias. We therefore decided to include the data using “vote counting”, a method recommended by the Cochrane Collaboration for summarizing data in systematic reviews, whenever only the direction of effect is presented and no further effect estimate is calculable [40]. This method summarizes the results of the studies by counting how many results show harm and how many show benefits from the intervention based on the direction of effect. That is, in this case, how many studies show increased or decreased risk after RAI administration. Confidence intervals (CIs) or p-values cannot be calculated when using vote counting. Therefore, it is crucial to emphasize that this method has serious limitations, because it does not consider the magnitude of the effect or the statistical precision of the effect estimate.

## Results

Ten articles were included in our analysis. The number of participants per study ranged from 895 to 148,215, the mean or median length of follow-up, from 5.9 to 16.2 years, and the mean or median age at DTC diagnosis, from 39.8 to 49.0 years. Overall, patient ages at diagnosis ranged from 2 to 100 years.

### Summary of individual studies

Rubino et al. [16] compiled data of 6841 participants from 10 hospitals in three countries and assessed the occurrence of SPM, of SHM, and of a dose–response relationship. They observed an elevated RR for SPM (1.2 [95% CI: 1.0–1.4]) and SHM (RR: 2.5 [95% CI: 1.0–7.4]) after RAI treatment, but no statistical significance was found. The RR increased with a cumulative administered ^131^I activity over 7.4 GBq, but again, no statistical significance was found. The ROB for all three outcomes of interest was judged to be serious due to missing data: it is noteworthy that approximately 20% of participants were lost to follow-up.

Khang et al. [20] included 2468 patients from one Korean hospital and investigated the occurrence of SPM and of a dose–response relationship. The authors found no significant effect of RAI treatment overall on SPM occurrence (OR: 1.14 [95% CI: 0.67–1.92]), but a significantly elevated OR with cumulative activities of 37 GBq ^131^I or more. Most patients received < 22.3 GBq cumulatively, and only a small group (*N* = 69) were treated with ≥ 37 GBq. ROBs for both outcomes were judged to be critical, as no information was reported about adjustment for important confounders like personal history of cancer or co-interventions, most notably, external radiotherapy. Furthermore, adjustment for gender and age at diagnosis remained unclear.

Hirsch et al. [19] examined the occurrence of SPM and of a dose–response relationship in 1792 patients from one Israeli institution and found no significantly increased risk for SPM after RAI treatment (HR: 1.27 [95% CI: 0.88–1.82]) and no sign of a dose–response relationship. The authors cross-matched their data with data from the Israel National Cancer Registry (INCR) to detect as many SPMs as possible. The ROB for both outcomes was rated as critical because antecedent malignancies and other cancer treatments, such as external radiotherapy, were not controlled for. Occurrence of SHM may have been underestimated because blood cancers are not reported to the INCR.

Silva-Vieira et al. [15] conducted a single-center study including 2031 participants from Portugal and detected a significantly elevated risk for SPM after RAI treatment (RR: 1.84 [95% CI: 1.02–3.31]) and a dose–response relationship, with significantly increased HRs from a cumulative ^131^I activity of 7.4 GBq onwards. The ROB for both outcomes was judged as moderate because we identified only minor sources of ROB.

Brown et al. [18] extracted data of 31,278 participants from the U.S. Surveillance, Epidemiology and End Results (SEER) database. From this study, we selected data of 9661 patients who had a minimum 3-year interval between RAI administration and SPM occurrence, contrary to the minimum 2-month latency time of the larger cohort. In our analysis, only cases between 1988 and 2002 were included, as before 1988, RAI exposure was not encoded as “radioisotope therapy” but as “other radiation”. Brown et al. presented standardized incidence ratios (SIRs) with the general population as the control cohort. The results showed an elevated SIR for participants treated with RAI (SIR: 1.23 [95% CI: 1.04–1.45]) but found no effect in participants not treated with RAI (SIR: 1.04 [95% CI: 0.9–1.2]). We included these data in our analysis using vote counting, because no direct comparison between these two groups was feasible. We judged the ROB in this study to be serious, because there was no information about controlling for external radiotherapy as a co-intervention.

Lang et al. [39] collected data from 895 patients from one institution in Hong Kong and calculated SIRs with the general population as controls. This analysis revealed an increased risk for SPM after RAI (SIR: 1.51 [95% CI: 1.14–1.96]), but not in the patients without exposure (SIR: 0.84 [95% CI: 0.36–1.66]). We also included these data using vote counting for the same reasons as enumerated for the Brown et al. study [18]. Furthermore, no significant dose–response relationship could be demonstrated. The ROB for both outcomes was rated as moderate.

Hakala et al. [22] analyzed data from 910 participants from two Finnish hospitals and controls matched for gender, age, and place of residence. The authors found no increased risk for SPM in irradiated (RR: 1.04 [95% CI: 0.83–1.32]) or non-irradiated patients (RR: 1.49 [95% CI: 0.96–2.30]). We included these data using vote counting for the above-mentioned reasons. Again, no evidence of a significant dose–response relationship was found. The ROB for both outcomes was judged as critical because personal history of cancer was not controlled for, and because approximately 30% of participants were lost to follow-up.

Teng et al. [21] extracted data of 20,235 participants from the Taiwanese National Health Insurance database and analyzed the occurrence of SPM and SHM and a dose–response relationship. All hazard ratios were calculated per 1.1 GBq. The authors found no significantly increased risk for SPM after RAI (HR: 1.01 [95% CI: 1.00–1.02]) but reported an elevated risk for leukemia after RAI (HR: 1.03 [95% CI: 1.02–1.04]). Furthermore, the data showed a dose–response relationship with increasing risk for SPM in subgroups with higher cumulative RAI activities. The ROB for all three outcomes was judged to be serious, most notably because all histological types of thyroid carcinoma were included.

Fallahi et al. [38] investigated a dose–response relationship in 973 patients from one Iranian institution. The results showed such a relationship, with a significantly elevated OR from a 40 GBq cumulative ^131^I activity onwards. The ROB was judged as serious because there was no adjustment for important confounders, most notably gender.

Molenaar et al. [41] extracted data from the SEER database and included 148,215 participants to analyze the occurrence of SHM after RAI treatment. Compared to the cohort presented by Brown et al. [18], this study is more up-to-date, but only SHMs after RAI were addressed. The results showed an increased risk for SHM (SIR: 1.30 [95% CI: 1.12–1.51]) after RAI treatment compared to surgery alone. The ROB was rated as moderate, as any source of ROB was minor.

**Fig. 2:**
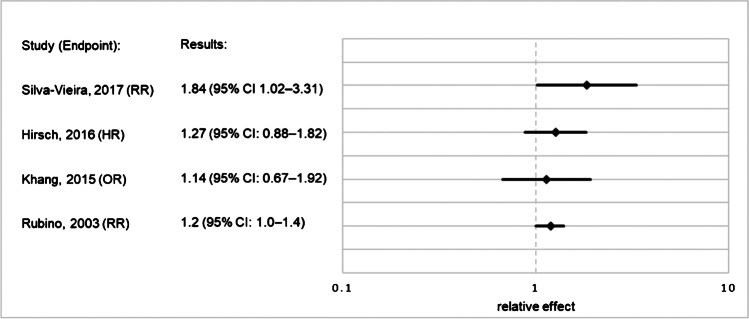
Relative effects for occurrence of SPM after RAI treatment vs. no RAI treatment in patients with DTC. Abbreviations: CI, confidence interval; DTC, differentiated thyroid cancer; HR, hazard ratio; OR, odds ratio; RAI, radioactive iodine; RR, relative risk; SPM, second primary malignancy

**Fig. 3:**
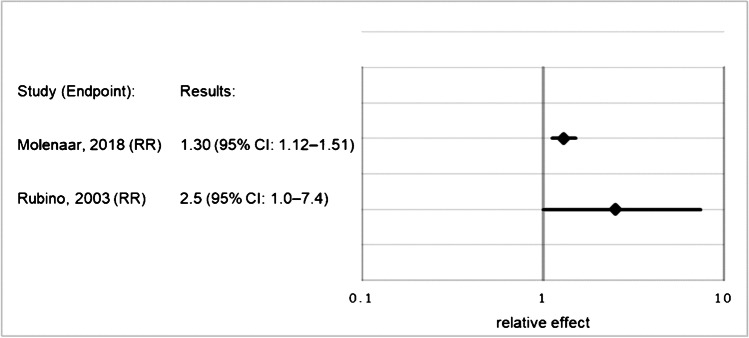
Relative effects for occurrence of SHM after RAI treatment vs. no RAI treatment in patients with DTC. Abbreviations: CI, confidence interval; DTC, differentiated thyroid cancer; RAI, radioactive iodine; RR, relative risk; SHM, secondary hematological malignancy

Further detailed information is presented in Online Resource 3. Relative effects for occurrence of SPM and SHM are presented in Figs. 2 and 3

### Quality of evidence

**Fig. 4:**
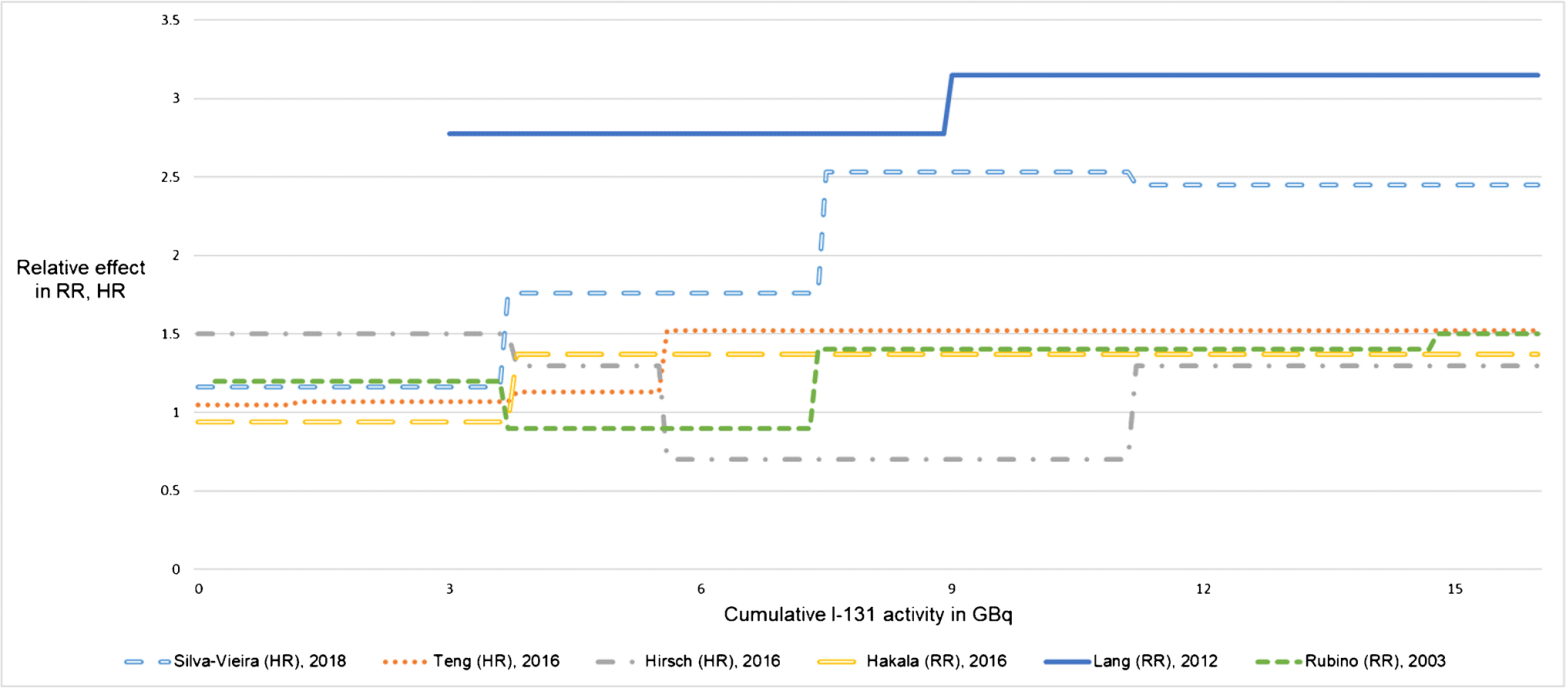
Relative effects (RR, HR) for occurrence of SPM after RAI treatment in patients with DTC according to cumulative RAI activity. Data from Fallahi et al. [38] and Khang et al. [20] are not shown, because there were only few participants in subgroups with high cumulative RAI activities, and we do not consider the given OR as comparable to the relative effects of the other studies. More comprehensive data are presented in Online Resource 3. Abbreviations: DTC, differentiated thyroid cancer; HR, hazard ratio; OR, odds ratio; RAI, radioactive iodine; RR, relative risk

The ROB data were used to apply the GRADE approach for evaluating QOE (Table 1, full information in Online Resource 4). For the risk of SPM, we acknowledged the ROB and the imprecision to be severe enough to downrate the QOE for both factors. We also found conclusive evidence indicating a dose–response relationship between RAI administration and SPM occurrence to uprate the evidence one level (Fig. 4). As the evidence from observational studies is preliminarily rated as low, the overall QOE for the outcome of SPM was rated as very low.

**Table 1:** Summary of findings table presenting the results of the GRADE synthesis and rating process. Table created with the GRADEpro Guideline Development Tool: GRADEpro GDT: GRADEpro Guideline Development Tool [Software]. McMaster University, 2020 (developed by Evidence Prime, Inc.). Available from gradepro.org. Table modified. Full information presented in Online Resource 4

Certainty assessment							Certainty
No of studies	Study design	Risk of bias	Inconsistency	Indirectness	Imprecision	Other considerations	
Second primary malignancies							
8	Observational studies	Serious	Not serious	Not serious	Serious	Dose–response gradient	⨁◯◯◯ VERY LOW
Secondary hematologic malignancies							
3	Observational studies	Not serious	Not serious	Not serious	Serious	Dose–response gradient	⨁⨁◯◯ LOW
Dose response gradient							
8	Observational studies	Serious	Not serious	Not serious	Serious	Dose–response gradient	⨁◯◯◯ VERY LOW

For the risk of SHM, we downrated the QOE by one level for imprecision, but then one level back up, as we found evidence indicating a dose–response relationship for SHM, as well [16, 21]. The overall QOE for the risk of SHM was judged as low.

For a dose–response relationship, we rated the ROB and the imprecision as substantial and downgraded one level for each factor. As the data indicated that a dose–response relationship was likely, we uprated the QOE one level. Overall, the QOE for a dose–response relationship between RAI treatment and SPM or SHM was judged as very low.

## Discussion

To our knowledge, this is the most up-to-date published systematic review investigating the attributable risk for SPM and SHM associated with RAI, and the first review of this topic to analyze the ROB and QOE regarding relative effects. The evidence suggests an increased risk for SPM and SHM after RAI administration, with a high probability of a dose–response relationship; however, due to substantial ROB and low QOE in the included studies, further research is required to substantiate these findings.

Using the ROBINS-I tool revealed many limitations in study design or execution. In most cases, adjustment for age at DTC diagnosis and sex was documented, and a personal history of cancer was often an exclusion criterion. Nonetheless, data concerning these and other potential confounders such as treatment of prior cancer or familial history of cancer were not always acquired or used to adjust the relative effect. For example, in the study of Fallahi et al., ORs lacked adjustment for gender, while in the study of Khang et al., factors for which the OR had been adjusted were not described [20, 38]. The omission of adjustments for treatments of antecedent malignancies may have represented a material baseline confounder, as those interventions might have had a carcinogenic effect.

Two papers revealed that approximately 20% or 30% of the participants were lost to follow-up, whereas only 8% and 12% of the participants had an SPM. This disparity led us to acknowledge a ROB due to missing data [16, 22].

The ROB due to selection of participants into a study was judged as moderate in every case, since only studies with a minimum latency of 1 year between DTC diagnosis and SPM occurrence were included in our analysis. The ROB due to measurement of outcomes was considered to be serious for the outcome “SHM” when the median or mean follow-up was < 3 years and for the outcome “SPM” when the follow-up was < 5 years. The ROB was judged to be moderate for the outcome “SHM” when the median or mean follow-up was < 5 years, and for the outcome “SPM” when the follow-up was < 8 years. To grade the ROB in this way was considered plausible because the carcinogenic effect of radiation only unfolds over years or even decades [26–28]. Therefore, we did not expect to miss a considerable number of SPMs during the first year of follow-up, while on the other hand, we were confident to minimize a screening bias in this way. Nevertheless, we may have overestimated the risk of SPM occurrence due to RAI therapy by using the conservative approach of a 12-month minimum latency period as an inclusion criterion. It is noteworthy that SHM seem to develop after a shorter latency period than do solid cancers [29]. External radiotherapy or chemotherapy for DTC were considered co-interventions, and resulted in a ROB due to deviation from intended intervention, if adjustment was missing.

The extracted data showed an effect of RAI plus surgery relative to surgery alone of 1.14 to 1.84 for the occurrence of SPM. Seven of 8 studies presented a higher risk in the exposed group compared with the non-exposed participants, but only one study presented a direct comparison between these groups that had statistically significant results [15]. Rubino et al. calculated an excess of 1.14 solid cancers and 0.08 leukemias per 10,000 person-years and GBq of administered ^131^I [16]. Overall, the absolute excess risk seems rather small, and this observation might, to some extent, explain why results are conflicting and this issue is still considered controversial.

Given the distinct thresholds or calculations of risk used by studies examining a dose–response relationship, we could not find a particular threshold of cumulative administered ^131^I activity leading to a significantly elevated risk of SPM. However, 7 of 8 studies presented higher relative effects for patients with higher cumulative activity compared with those receiving lower ^131^I activities. These results strongly suggest a dose–response relationship.

Only one included study [16] specifically analyzed SPM in patients given RAI as children or young adults. Recent studies found a high prevalence of cancer predisposition syndromes in childhood cancer survivors developing SPM [42, 43]. Further research is needed to investigate the impact of genetic susceptibility on the one hand, and of radiation exposure or chemotherapy in relation to age on the other. As randomization was lacking in every study, we cannot exclude genetic susceptibility affecting our overall results as well.

The most recent meta-analysis published before our critical review presented unadjusted RR (0.98 [95% CI: 0.76–1.27]; *N* = 10 studies) and adjusted RR (1.16 [95% CI: 0.97–1.39]; *N* = 6 studies) of a pooled random-effects analysis for the outcome of SPM [23]. Heterogeneity was statistically evaluated using the I^2^ measure and was found to be relevant (I^2^ = 85.99 for the unadjusted RR, I^2^ = 56.26 for the adjusted RR). These findings are in accordance with our results. Furthermore, the authors utilized a funnel-plot to reveal indications of a publication bias. We could not evaluate publication bias, as we did not analyze pooled data of included studies. As a result, we may have overestimated QOE, since that form of bias is grounds for downgrading QOE when using the ROBINS-I tool.

Another review [13], published in 2015, included 6 studies, of which 5 were included in our analysis, but we could not reproduce that review’s findings regarding ROB and QOE. Whereas Clement et al. judged the QOE for SPM after RAI and for a dose–response relationship as “moderate”, we rated the QOE as “very low” for both outcomes. These differences might, to some extent, be explained by the use of different ROB rating tools. Furthermore, Clement et al. did not investigate the ROB regarding relative effect measures, but that regarding SPM incidence after RAI administration.

The main limitations concerning any kind of synthesis of results originate from the lack of comparability between studies. Cohorts may present vast heterogeneity internally and comparatively based on exposure to lifestyle factors (e.g., diet, overweight) or carcinogens (e.g., alcohol, tobacco), ethnicity, or different screening practices. These confounders were not routinely recorded in most studies. As the baseline incidence of cancers in general and cancers of specific sites differ widely between countries and regions, those confounders also need to be considered to achieve better comparability between studies. This heterogeneity between cohorts is also reflected by a substantial variation of the crude incidence of SPM occurrence (1.1–12.0%) [22, 38] and can only, to some extent, be explained by different sample size and length of follow-up. Additionally, it is crucial to include appropriate control groups; 32 of 59 papers ineligible for our analysis were excluded due to their comparing only RAI-treated patients, and not their counterparts receiving surgery alone, with the general population.

ROBINS-I has limitations that must be considered when interpreting our results. First, the tool is not designed to present distinct results regarding the magnitude or likelihood of particular potential biases. The magnitude of bias is reflected only in the overall ROB judgment. Raters must elaborate a judgment for each ROBINS-I domain, and then sum those ratings into an overall ROB judgment. For example, if the rater is rather confident that a serious bias exists in two or more ROB domains, the overall ROB judgment might be “critical”, while one would refrain from downrating the overall ROB in case of lesser certainty. Unfortunately, this aspect of the ROBINS-I assessment is less transparent.

In summary, most included studies suggest increased risk for SPM and SHM after RAI administration, and an association between increased cumulative administered activities of RAI and the risk of SPM. Nonetheless, given the high ROB and low QOE of reports to date, further research is required to substantiate these findings. Our analysis revealed three problems that future studies should address: (1) ROB due to a variety of limitations, (2) imprecision of reported results, and (3) inappropriate control groups. To mitigate these shortcomings, researchers should perform adequate statistical adjustment, i.e., control more comprehensively for relevant factors. Additionally, investigators should ensure extensive follow-up duration, and reduce proportions of participants who are lost to follow-up. Moreover, future studies should include larger cohorts and appropriate control groups comprising patients with DTC without RAI exposure. Lastly, future studies should investigate which patients profit from RAI treatment and which ^131^I activities should be used. For example, mounting evidence suggests that patients with clonal hematopoiesis of indeterminate potential have, per se, a multiple times higher risk of secondary myeloid malignancy or leukemia [44, 45], which increases further after cytotoxic therapies [46]. Patient risk stratification based on histology and clinical factors may be augmented by “molecular theragnostics” [47]. Additionally, pre-therapeutic dosimetry may be useful for ^131^I dose adjustment [48, 49]. Both approaches facilitate individualized therapy and may reduce adverse effects, while simultaneously ensuring patient-relevant outcomes.

Meanwhile, in view of the low or very low QOE and the apparently small relative effect of RAI on SPM occurrence, we suggest that use of RAI, in which much stronger evidence has demonstrated to be effective treatment for DTC [50–52], not be restricted based on SPM or SHM risk.

## Supplementary Information

Below is the link to the electronic supplementary material.

Supplementary file1 (PDF 146 KB)

Supplementary file2 (PDF 181 KB)

Supplementary file3 (PDF 159 KB)

Supplementary file4 (PDF 557 KB)
