# Supplementary material for: Second primary malignancies induced by radioactive iodine treatment of differentiated thyroid carcinoma — a critical review and evaluation of the existing evidence
Source: Eur J Nucl Med Mol Imaging. 2022 Mar 23;49(9):3247–56. doi: 10.1007/s00259-022-05762-4 (PMC9250458; doi:10.1007/s00259-022-05762-4)
Supplement: Supplementary file 1 — Supplementary file1 (PDF 146 KB) [file 259_2022_5762_MOESM1_ESM.pdf]

Online Resource 1: Queries used to perform an extensive electronic literature search. Also presented are the date of the queries and the number of retrieved articles in total and after removal of duplicate papers.

Pubmed (NCBI)

(((second primary cancer[MeSH Terms]) OR (((((((((((second primary cancer) OR second primary carcinoma) OR second primary malignancy) OR second primary neoplasm) OR second primary cancers) OR second primary carcinomas) OR second primary malignancies) OR second primary neoplasms) OR subsequent cancer) OR subsequent carcinoma) OR subsequent malignoma) OR second primary malignoma) OR subsequent neoplasms))) AND (((((((((thyroid cancer) OR thyroid neoplasm) OR thyroid carcinoma) OR thyroid malignancy) OR thyroid malignancies) OR thyroid cancers) OR thyroid carcinomas) OR thyroid neoplasms)) OR thyroid cancer[MeSH Terms])

Originally searched: 3.5.2020

Updated: 13.12.2020

(Overall: 4983 articles)

Ovid MEDLINE, MEDLINE Epub Ahead of Print and In-Process & Other Non-Indexed Citations

((Thyroid cancer or thyroid neoplasms or thyroid malignancy) **af**).af. AND ((second primary cancer or second primary neoplasms or second primary malignancies or second primary malignancy or second primary carcinoma or subsequent carcinoma or subsequent cancer or subsequent malignancy or subsequent malignancies) af).af.

Originally searched: 3.5.2020

Updated: 13.12.2020

(Overall: 148 articles)

Cochrane Central Register of Controlled Trials (CENTRAL)

"("second primary cancer"):ti,ab,kw OR ("second primary malignancies"):ti,ab,kw OR ("second primary tumor"):ti,ab,kw AND ("thyroid cancer"):ti,ab,kw (Word variations have been searched)" (Word variations have been searched)

Searched: 3.5.2020

Updated: 13.12.2020

(Overall 284 articles)

Total articles, including duplicate papers: n=5415

Total articles after removal of duplicate papers: n=5269

Article Title: Second primary malignancies induced by radioactive iodine treatment of differentiated thyroid carcinoma – a critical review and evaluation of the existing evidence

Journal Name: European Journal of Nuclear Medicine and Molecular Imaging

Authors:

Maximilian J. Reinecke, Department of Nuclear Medicine, University Hospital Marburg, Marburg, Germany

Gerrit Ahlers, Department of Nuclear Medicine, University Hospital Marburg, Marburg, Germany

Andreas Burchert, Department of Internal Medicine, Hematology, Oncology and Immunology, University Hospital Marburg, Marburg, Germany

Friederike Eilsberger, Department of Nuclear Medicine, University Hospital Marburg, Marburg, Germany

Glenn D. Flux, Department of Physics, Royal Marsden Hospital and Institute of Cancer Research, Sutton, United Kingdom

Robert J. Marlowe, Spencer-Fontayne Corporation, Jersey City, NJ 07304-1901, USA

Hans-Helge Mueller, Institute for Medical Bioinformatics and Biostatistics, Philipps University of Marburg, Marburg, Germany

Christoph Reiners, Department of Nuclear Medicine, University Hospital Wuerzburg, Wuerzburg, Germany

Fenja Rohde, Department of Nuclear Medicine, University Hospital Marburg, Marburg, Germany

Hanneke M. van Santen, Department of Pediatrics, Wilhelmina Children's Hospital, University Medical Center Utrecht, Utrecht, The Netherlands; Princess Máxima Center for Pediatric Oncology, Utrecht, The Netherlands

Markus Luster (Corresponding Author), Department of Nuclear Medicine, University Hospital Marburg, Marburg, Germany

luster@med.uni-marburg.de
