# Supplementary material for: Second primary malignancies induced by radioactive iodine treatment of differentiated thyroid carcinoma — a critical review and evaluation of the existing evidence
Source: Eur J Nucl Med Mol Imaging. 2022 Mar 23;49(9):3247–56. doi: 10.1007/s00259-022-05762-4 (PMC9250458; doi:10.1007/s00259-022-05762-4)
Supplement: Supplementary file 2 — Supplementary file2 (PDF 181 KB) [file 259_2022_5762_MOESM2_ESM.pdf]

Online Resource 2: Some included articles provided different data about the risk of second primary malignancies after radioactive iodine treatment. This appendix provides information about data not included in our analysis and the respective rationale. Abbreviations: aHR, adjusted hazard ratio; CI, confidence interval; HR, hazard ratio; O/E, observed/expected cases; OR, odds ratio; RAI, radioactive iodine; RR, relative risk; SHM, second hematologic malignancy; SIR, standardized incidence ratio; SPM, second primary malignancy.

Study/ Outcome/Citation	Extracted data	Data not extracted and explanation why
Rubino, 2003/<br>SPM<br>[16]	Reference: Table 2, Cancer site: “At least one cancer”<br>RR, stratified by study group and adjusted for external radiotherapy: I-131 vs. no I-131 for the outcome SPM<br>RR: 1.2 (95% CI: 1.0–1.4)	
Rubino, 2003/<br>SHM<br>[16]	Reference: Table 2, Cancer Site: “Leukaemia”<br>Relative Risk (RR), stratified by study group and adjusted for external radiotherapy: I-131 vs. no I-131 for the outcome leukemia<br>RR: 2.5 (95% CI: 1.0–7.4)	
Rubino, 2003/<br>Dose-response relationship<br>[16]	Reference: Table 3, Type of SPM: “Solid cancers”<br>RR: The occurrence of second primary solid cancers depending on the cumulative RAI activity administered, patients treated with external radiotherapy excluded.<br>≤0.2 GBq: 1.0 (reference)<br>>0.2–3.6 GBq: 1.2 (95% CI: 0.9–1.5)<br>3.7-7.3 GBq: 0.9 (95% CI: 0.7–1.2)<br>7.4-14.7 GBq: 1.4 (95% CI: 1.0–2.1)<br>≥14.8 GBq: 1.5 (95% CI: 0.8–2.6)	

Study/ Outcome/Citation	Extracted data	Data not extracted and explanation why
Brown, 2008/<br>SPM<br>[18]	Reference: Table 6<br>SIR = O/E, 36-month latency exclusion, 1988-2002, SPM all sites:<br>No radiotherapy: 1.04 (95% CI: 0.9–1.2)<br>Radioisotopes: 1.23 (95% CI: 1.04–1.45)	We included data from 9,661 patients with a minimum 3-year latency period until SPM occurrence and a diagnosis from 1988 on, because before this period, RAI was not specifically encoded into the medical record. Data from 30,278 patients with a minimum 2-month latency period until SPM occurrence and a TC diagnosis between 1973 and 2002 would have been available. (Tables 2/3) We decided not to include this larger cohort, because these data would have a higher risk of bias.
Fallahi, 2011/<br>Dose-response relationship<br>[38]	Reference: Table 4<br>Odds Ratios (OR) of SPM with increasing cumulative activity of RAI<br><10 GBq: 1.00 (Reference)<br>10-20 GBq: 3.11 (95% CI: 0.24–39.83)<br>20-30 GBq: 9.29 (95% CI: 0.69–125.01)<br>30-40 GBq: No SPM cases<br>40-50 GBq: 113.42 (95% CI: 8.60-1495.64)<br>≥50 GBq: 122.90 (95% CI: 5.56-2716.93)	.
Lang, 2012/<br>SPM<br>[39]	Reference: Table IV<br>SIR:<br>RAI(+) group: 1.51 (95% CI: 1.14-1.96)<br>RAI(-) group: 0.84 (95% CI: 0.36-1.66)	
Lang, 2012/<br>Dose-response relationship<br>[39]	Reference: Table III<br>RR, Cox proportional hazards analysis, cumulative RAI activity [GBq]<br>None: Reference<br>3.0–8.9 GBq: 2.777 (95% CI: 1.089–7.145)<br>>9.0 GBq: 3.149 (95% CI: 0.645–12.816)	

Study/ Outcome/Citation	Extracted data	Data not extracted and explanation why
Hakala, 2012/<br>SPM<br>[22]	RR (No-RAI vs. controls): 1.49 (95% CI: 0.96–2.30) - Reference: Table 2<br>RR (RAI vs. Controls): 1.04 (95% CI: 0.83-1.32) - Reference: Table 4	In Table 2, a "Multivariable analysis" was conducted (RR: 1.12 [CI: 0.91-1.38]), but RAI-treated patients seem to be compared to their controls, not to not irradiated patients.<br>All in all, for our outcome of interest, there were no more up-to-date or more extensive results presented in this paper.
Hakala, 2012/<br>Dose-response relationship<br>[22]	Reference: Table 2<br>(Patients vs. controls) as Rate Ratio for subgroups according to their cumulative RAI activity:<br>≤3.7 GBq: 0,94 (95% CI: 0.70–1.25)<br>>3.7 GBq: 1.37 (95% CI: 0.90–2.09)	
Khang, 2015/<br>SPM<br>[20]	Reference: Table 3<br>OR [RAI(+) vs. RAI(-)]: 1.14 (95% CI: 0.672–1.915)	
Khang, 2015/<br>Dose-response<br>Relationship<br>[20]	Reference: Table 3<br>OR [RAI(+) in various activities vs. RAI(-)]:<br>1.1-5.55 GBq: 0.87 (95% CI: 0.469–1.620)<br>5.56-22.2 GBq: 0.67 (95% CI: 0.272–1.659)<br>22.3-36.9 GBq: 2.04 (95% CI: 0.477–8.696)<br>≥37.0 GBq: 5.54 (95% CI: 2.635–11.634)	
Hirsch, 2016/<br>SPM<br>[19]	Reference: p.1113, last paragraph of "Results"<br>Hazard Ratio (HR) "for SPM in patients after first RAI treatment compared to patients with no RAI treatment, adjusted for age and sex": 1.27 [95% CI: 0.88–1.82]	1,943 patients were included in the paper, but only 1,792 had ≥2 years of follow-up and were therefore included in our analysis concerning the association between RAI therapy and SPM occurrence.

Study/ Outcome/Citation	Extracted data	Data not extracted and explanation why
Hirsch, 2016/<br>Dose-response relationship<br>[19]	Reference: Table 5<br>Hazard Ratio (HR) depending on cumulative "dose" [activity] [in mCi]:<br>1–100 [0.037–3.7 GBq] : 1.5 (95% CI: 0.99–2.3)<br>101–150 [3.737–5.55 GBq]: 1.3 (95% CI: 0.8–2.1)<br>151–299: [5.587–11.063 GBq] 0.7 (95% CI: 0.3–1.5)<br>>300 [11.1 GBq]: 1.3 (95% CI: 0.8–2.4)	1,943 patients were included in the paper, but only 1,792 had ≥2 years of follow-up and were therefore included in our analysis concerning the association between RAI therapy and SPM occurrence
Teng, 2016/<br>SPM<br>[21]	Reference: Supplementary tables, Table 4<br>Adjusted HR (aHR) of the cumulative RAI dose per 30 mCi [1.11 GBq] increase; patients with history of external beam radiation or chemotherapy were excluded<br>aHR: 1.01 (95% CI: 1.00-1.02)	Supplementary Table 2 and 3B: almost similar results presented, but it remains unclear for which confounders the HRs were adjusted.<br>Table 5 and 6: patients with history of external radiotherapy or chemotherapy were not excluded.
Teng, 2016/<br>SHM<br>[21]	Reference: Supplementary tables, Table 4<br>aHR of cumulative RAI dose per 30 mCi [1.11 GBq] increase; patients receiving external beam radiation or chemotherapy were excluded<br>Leukemia aHR: 1.03 (95% CI: 1.02–1.04)<br>Non-Hodgkin lymphoma aHR: 0.86 (95% CI: 0.73-1.00)	Supplementary Table 2 and 3B: almost similar results presented, but it remains unclear for which confounders the HRs were adjusted.<br>Table 5 and 6: patients with history of external radiotherapy or chemotherapy were not excluded.
Teng, 2016/<br>Dose-response relationship<br>[21]	Reference: Supplementary tables, Table 4<br>aHR according to the cumulative radioactive activity; patients receiving external beam radiation or chemotherapy were excluded<br>1–30 mCi [0.037–1.11 GBq] aHR: 1.05 (95% CI: 0.81–1.35)<br>30–100 mCi [1.11–3.7 GBq] aHR: 1.07 (95% CI: 0.85–1.34)<br>100–150 mCi [3.7– 5.55 GBq] aHR: 1.13 (95% CI: 0.851.50)<br>>150 mCi aHR [>5.55 GBq]: 1.52 (95% CI: 1.19–1.95)	Table 6: patients with external radiotherapy and chemotherapy were not excluded.
Silva-Vieira, 2017/<br>SPM<br>[15]	Reference: Table 3<br>RR of "Treatment with radioiodine – yes versus no": 1.84 (95% CI: 1.02–3.31)	

Study/ Outcome/Citation	Extracted data	Data not extracted and explanation why
Silva-Vieira, 2017/<br>Dose-response relationship<br>[15]	Reference: Table 4<br>HR – Cox regression model (no competitive risk) – Cumulative activity each vs. 0 mCi [0 GBq]<br><100 mCi [<3.7 GBq]: 1.16 (95% CI: 0.44–3.06)<br>100-199 mCi [3.7–7.363 GBq]: 1.76 (95% CI: 0.95–3.28)<br>200-299 mCi [7.4–11.063 GBq]: 2.53 (95% CI: 1.21–5.30)<br>≥300 mCi [≥11.1 GBq]: 2.45 (95% CI: 1.12–5.36)	
Molenaar, 2018/<br>SHM<br>[41]	Reference: Table 3<br>RR for all SHM combined, additional risk from RAI<br>RR: 1.30 (95% CI: 1.12–1.51)	Reference: Table 2<br>HR for all SHM combined<br>HR: 1.43 (95% CI: 1.20-1.69)<br>We decided to prefer RR, whenever available, as results, for better comparability with other included studies.

Article Title: Second primary malignancies induced by radioactive iodine treatment of differentiated thyroid carcinoma – a critical review and evaluation of the existing evidence

Journal Name: European Journal of Nuclear Medicine and Molecular Imaging

Authors:

Maximilian J. Reinecke, Department of Nuclear Medicine, University Hospital Marburg, Marburg, Germany

Gerrit Ahlers, Department of Nuclear Medicine, University Hospital Marburg, Marburg, Germany

Andreas Burchert, Department of Internal Medicine, Hematology, Oncology and Immunology, University Hospital Marburg, Marburg, Germany

Friederike Eilsberger, Department of Nuclear Medicine, University Hospital Marburg, Marburg, Germany

Glenn D. Flux, Department of Physics, Royal Marsden Hospital and Institute of Cancer Research, Sutton, United Kingdom

Robert J. Marlowe, Spencer-Fontayne Corporation, Jersey City, NJ 07304-1901, USA

Hans-Helge Mueller, Institute for Medical Bioinformatics and Biostatistics, Philipps University of Marburg, Marburg, Germany

Christoph Reiners, Department of Nuclear Medicine, University Hospital Wuerzburg, Wuerzburg, Germany

Fenja Rohde, Department of Nuclear Medicine, University Hospital Marburg, Marburg, Germany

Hanneke M. van Santen, Department of Pediatrics, Wilhelmina Children's Hospital, University Medical Center Utrecht, Utrecht, The Netherlands; Princess Máxima Center for Pediatric Oncology, Utrecht, The Netherlands

Markus Luster (Corresponding Author), Department of Nuclear Medicine, University Hospital Marburg, Marburg, Germany

luster@med.uni-marburg.de
