# Supplementary material for: Second primary malignancies induced by radioactive iodine treatment of differentiated thyroid carcinoma — a critical review and evaluation of the existing evidence
Source: Eur J Nucl Med Mol Imaging. 2022 Mar 23;49(9):3247–56. doi: 10.1007/s00259-022-05762-4 (PMC9250458; doi:10.1007/s00259-022-05762-4)
Supplement: Supplementary file 3 — Supplementary file3 (PDF 159 KB) [file 259_2022_5762_MOESM3_ESM.pdf]

Online Resource 3: Presented are detailed data about included papers (first author, year of publication), extracted data (country of the included cohort, relative effect endpoint and results, crude incidence of SPMs, follow-up period, number of participants, rate of patients treated with RAI) and the evaluated risk of bias of each study. Abbreviations: aHR, adjusted hazard ratio; CI, confidence interval; HR, hazard ratio; IQR, interquartile range; OR, odds ratio; RAI, radioactive iodine; ROB, risk of bias; RR, relative risk; SEER, Surveillance, Epidemiology, and End Results Program; SHM, secondary hematologic malignancy; SIR, standardized incidence ratio; SPM, second primary malignancy.

Occurrence of SPMs in patients treated with RAI versus without RAI							
**Study/ Citation**	**Cohort/Country**	**Risk of bias**	**Relative effect endpoint**	**Results**	**Crude Incidence of SPM**	**Follow-up period**	**Participants (rate of RAI treatment)**
Silva-Vieira, 2017 [15]	Portugal	Moderate	**RR** of "treatment with radioiodine – yes versus no"	1.84 (95% CI: 1.02–3.31)	Total: 6.4%<br>RAI(+): 6.9%<br>RAI(-): 4.8%	Median: 8.8y<br>Range: 5.0-17.0y	2,031<br>(77%)
Hirsch, 2016 [19]	Israel	Critical	**HR** "for SPM in patients after first RAI treatment compared to patients with no RAI treatment, adjusted for age and sex"	1.27 (95% CI: 0.88–1.82)	Total: 9.3%	Mean ± SD:<br>9.3 ± 9.6y	1,792<br>(81.9%)
Khang, 2015 [20]	Korea	Critical	**OR** [RAI(+) vs. RAI(-)]	1.14 (95% CI: 0.672–1.915)	Total: 2.5%	Median: 7.0y<br>Range: 1.0-33.0y	2,468<br>(56.6%)
Rubino, 2003 [16]	Sweden/Italy/ France	Serious	**RR,** stratified by study group and adjusted for external radiotherapy: I-131 vs. no I-131	1.2 (95% CI: 1.0-1.4)	Total: 8.4%	Mean: 13y<br>Range: 2-55y	6,841<br>(62%)
Teng, 2016 [21]	Taiwan	Serious	**aHR** of the cumulative RAI activity per 30 mCi [1.11 GBq] increase; patients receiving external beam radiation or chemotherapy were excluded	1.01 (95% CI: 1.00-1.02)	Total: 3.4%	Median: 5.9y<br>IQR: 3.1-9.9y	20,235<br>(58.3%)
Hakala, 2016 [22]	Finland	Critical	Two numerical results were analyzed to compare the two **rate ratios (RR)** for no RAI vs. controls and RAI vs. controls	No RAI vs controls: 1.49 (95% CI: 0.96–2.30)<br>RAI vs. controls: 1.04 (95% CI: 0.83–1.32)	Total: 12%	Mean ± SD:<br>16.2 ± 6.9y	910<br>(81%)
Lang, 2012 [39]	Hong Kong	Moderate	**SIR**	RAI(+) group: 1.51 (95% CI: 1.14–1.96)<br>RAI(-) group: 0.84 (95% CI: 0.36–1.66)	Total: 7.2%	Median: 7.8y<br>Range: 2.0-47.6y	895<br>(71.8%)
Brown, 2008 [18]	SEER, U.S.	Serious	**SIR**, <36-month latency exclusion, 1988-2002 exclusion, all sites of SPM	No radiotherapy: 1.04 (95% CI: 0.9–1.2)<br>"Radioisotopes": 1.23 (95% CI: 1.04–1.45)	Total: 7.1%	Median: 8.6y<br>Range: 0.2-29.9y	30,278<br>(44.0%)

Dose-response relationship – Occurrence of SPM according to cumulative RAI activity							
**Study**	**Cohort/Country**	**Overall RoB**	**Relative effect endpoint(s)**	**Results**	**Crude Incidence of SPM**	**Follow-up period**	**Participants (rate of RAI treatment)**
Silva-Vieira, 2017 [15]	Portugal	Moderate	**HR** – Cox regression model (no competitive risk) – Cumulative activity each activity subgroup vs. 0 mCi [0 GBq]	<100 mCi [<3.7 GBq]: 1.16 (95% CI: 0.44–3.06)<br>100–199 mCi [3.7–7.363 GBq]: 1.76 (95% CI: 0.95–3.28)<br>200–299 mCi [7.4–11.063 GBq]: 2.53 (95% CI: 1.21–5.30)<br>≥300 mCi [≥11.1 GBq]: 2.45 (95% CI: 1.12–5.36)	Total: 6.4%<br>RAI(+): 6.9%<br>RAI(-): 4.8%	Median: 8.8y<br>Range: 5.0-17.0y	2,031<br>(77%)
Teng, 2016 [21]	Taiwan	Serious	**aHR** according to the cumulative radioactive activity category; patients receiving external beam radiation or chemotherapy were excluded	1-30 mCi [0.037–1.11GBq] : 1.05 (95% CI: 0.81–1.35)<br>>30–100 mCi [>1.11–3.7 GBq] : 1.07 (95% CI: 0.85–1.34)<br>>100–150 mCi [>3.7–5.55 GBq] : 1.13 (95% CI: 0.85–1.50)<br>>150 mCi [>5.55 GBq] : 1.52 (95% CI: 1.19 - 1.95)	Total: 3.4%	Median: 5.9y<br>IQR: 3.1-9.9y	20,235<br>(58.3%)
Hirsch, 2016 [19]	Israel	Critical	**HR** depending on cumulative activity category [in mCi]	1–100 [0.037–3.7 GBq]: 1.5 (95% CI: 0.99–2.3)<br>101–150 [3.737–5.55GBq]: 1.3 (95% CI: 0.8–2.1)<br>151–299 [5.587–11.063 GBq]: 0.7 (95% CI: 0.3–1.5)<br>≥300 [≥11.1 GBq]: 13 (95% CI: 0.8–2.4)	Total: 9.3%	Mean ± SD:<br>9.3 ± 9.6y	1,792<br>(81.9%)
Hakala, 2016 [22]	Finland	Critical	(Patients vs. controls) as **Rate ratio (RR)** for subgroups according to their cumulative RAI activity	≤3.7 GBq: 0.94 (95% CI: 0.70–1.25)<br>>3.7 GBq: 1.37 (95% CI: 0.90–2.09)	Total: 12%	Mean ± SD:<br>16.2 ± 6.9y	910<br>(81%)
Khang, 2015 [20]	Korea	Critical	**OR** [RAI(+) according to cumulative activity group vs. RAI(-)]	1.1–5.55 GBq: 0.87 (95% CI: 0.469–1.620)<br>5.56–22.2 GBq: 0.67 (95% CI: 0,272–1,659)<br>22.3–36.9 GBq: 2.04 (95% CI: 0.477–8.696)<br>≥37.0 GBq: 5.54 (95% CI: 2.635-11.634)	Total: 2.5%	Median: 7.0y<br>Range: 1.0-33.0y	2,468<br>(56.6%)

Lang, 2012 [39]	Hong Kong	Moderate	**RR,** Cox proportional hazards analysis, cumulative RAI activity [GBq]	None: Reference<br>3.0–8.9 GBq: 2.777 (95% CI: 1.089–7.145)<br>>9.0 GBq: 3.149 (95% CI: 0.645–12.816)	Total: 7.2%	Median: 7.8y<br>Range: 2.0-47.6y	895 (71.8%)
Fallahi, 2011 [38]	Iran	Serious	**OR** of SPM with increasing cumulative activity of RAI	<10 GBq: 1.00 (reference)<br>10–20 GBq: 3.11 (95% CI: 0,24-39,83)<br>20–30 GBq: 9.29 (95% CI: 0,69-125,01)<br>30–40 GBq: No SPM cases<br>40–50 GBq: 113.42 (95% CI: 8,60-1495,64)<br>≥50 GBq: 122.90 (95% CI: 5.56-2716.93)	Total: 1.1%	Median: 6y<br>Mean: 7.5y<br>Range: 3-26y	973 (100%)
Rubino, 2003 [16]	Sweden/ Italy/ France	Serious	**RR:** The occurrence of second primary solid cancers depending on the cumulative RAI activity administered, patients treated with external radiotherapy excluded.	≤0.2 GBq: 1.0 (reference)<br>0.2–3.6 GBq: 1.2 (95% CI: 0.9–1.5)<br>3.7–7.3 GBq: 0.9 (95% CI: 0.7–1.2)<br>7.4–14.7 GBq: 1.4 (95% CI: 1.0–2.1)<br>≥14.8 GBq: 1.5 (95% CI: 0.8–2.6)	Total: 8.4%	Mean: 13y<br>Range: 2-55y	6,841 (62%)

Occurrence of SHM in patients treated with RAI versus without RAI							
**Study**	**Cohort/Country**	**Risk of Bias**	**Measure**	**Data**	**Crude Incidence**	**Follow-up period**	**Participants (RAI rate)**
Molenaar, 2018 [41]	SEER, U.S.	Moderate	**SIR** for all SHMs combined, additional risk from RAI	1.30 (95% CI: 1.12–1.51)	Total: 0.53%	Median: 6.6y<br>IQR: 3.3-11.2y	148,215 (47%)
Rubino, 2003 [16]	Sweden/Italy/ France	Serious	**RR**, stratified by study group and adjusted for external radiotherapy: I-131 vs. no I-131 for the outcome leukemia	2.5 (95% CI: 1.0–7.4)	Total: 18/6,841 (0.26%)	Mean: 13y<br>Range: 2-55y	6,841 (62%)
Teng, 2016 [21]	Taiwan	Serious	**aHR** of cumulative RAI activity **per 30 mCi [1.1 GBq]** increase; patients receiving external beam radiation or chemotherapy were excluded	Leukemia: 1.03 (95% CI: 1.02–1.04)<br>Non-Hodgkin Lymphoma: 0.86 (95% CI: 0,73-1,00)	Leukemia: 19/20,235 (0.09%)<br>Non-Hodgkin Lymphoma: 26/20,235 (0.13%)	Median: 5.9y<br>IQR: 3.1-9.9y	20,235 (58.3%)
