# Supplementary material for: Second primary malignancies induced by radioactive iodine treatment of differentiated thyroid carcinoma — a critical review and evaluation of the existing evidence
Source: Eur J Nucl Med Mol Imaging. 2022 Mar 23;49(9):3247–56. doi: 10.1007/s00259-022-05762-4 (PMC9250458; doi:10.1007/s00259-022-05762-4)
Supplement: Supplementary file 4 — Supplementary file4 (PDF 557 KB) [file 259_2022_5762_MOESM4_ESM.pdf]

Online Resource 4: Summary of findings table presenting the results of the GRADE synthesis and rating process. Table created with the GRADEpro Guideline Development Tool: GRADEpro GDT: GRADEpro Guideline Development Tool [Software]. McMaster University, 2020 (developed by Evidence Prime, Inc.). Available from gradepro.org. Abbreviations: CI, confidence interval; HR, hazard ratio; OR, odds ratio; RAI, radioactive iodine; ROB, risk of bias; RR, relative risk; SHM, secondary hematologic malignancy; SPM, second primary malignancy.

Question: Occurrence of second primary malignancies and hematologic malignancies after radioactive iodine therapy for differentiated thyroid carcinoma

Certainty assessment							Impact	Certainty
**№ of studies**	**Study design**	**Risk of bias**	**Inconsistency**	**Indirectness**	**Imprecision**	**Other considerations**		
**Second Primary Malignancies**								
8	observational studies	serious [a]	not serious	not serious	serious [b]	dose-response gradient	Relative effect range: (RR, OR, HR) 1.14–1.84<br><br>Vote counting: 7/8 studies showed an increased risk for SPM after RAI therapy compared to patients not exposed to RAI. 1/8 study showed an increased risk for SPM in patients without RAI therapy compared to patients who received RAI therapy.<br><br>[c]	⨁◯◯◯<br>VERY LOW
**Secondary Hematologic Malignancies**								
3	observational studies	not serious [d]	not serious	not serious	serious [e]	dose-response gradient	Relative effect range: (RR) 1.30-2.5<br><br>Vote counting: 3/3 studies showed an increased risk for SHM after RAI therapy compared to patients not exposed to RAI.	⨁⨁◯◯<br>LOW
**Dose Response Gradient**								
8	observational studies	serious [f]	not serious	not serious	serious [g]	dose-response gradient	The data indicate a dose-response gradient.<br><br>Vote counting: Comparing the groups with the least and the greatest cumulative activity of RAI, 7/8 studies found an increased relative effect.	⨁◯◯◯<br>VERY LOW

Explanations:

a. We judged the risk of bias using the ROBINS-I Tool. We judged the overall ROB as serious because only 2/8 studies have a moderate ROB, while 3/8 have a serious ROB, and 3/8 have a critical ROB.

b. The confidence intervals of most studies show a wide range and often include the relative effect under and above 1.0.

c. Data of 4 studies were included in this analysis. Statistical significance was demonstrated in 1/4 studies. [Silva-Vieira et al.[15]]

d. We judged the ROB using the ROBINS-I Tool. We judged the overall ROB not as serious because 2 studies were judged as having a serious ROB, but the largest and most up-to-date study was judged as having a moderate ROB.

e. Teng et al. presented the data as HR per 30 mCi (1.11 GBq). These results are not directly comparable with those of the other two studies. Rubino et al.[16] presented results with wide confidence intervals. All in all, the data is based on rather few cases.

f. We judged the ROB using the ROBINS-I Tool. We judged the overall ROB as serious because 2/8 studies have a moderate ROB, 3/8 studies have a serious ROB and 3/8 have a critical ROB. Whereas studies with a moderate or critical ROB analyzed rather small cohorts, two of the studies with a serious ROB analyzed larger cohorts (*N*=6841, *N*=20235).

g. The confidence intervals of most studies showed a wide range and many studies analyzed rather few participants.

Article Title: Second primary malignancies induced by radioactive iodine treatment of differentiated thyroid carcinoma – a critical review and evaluation of the existing evidence

Journal Name: European Journal of Nuclear Medicine and Molecular Imaging

Authors:

Maximilian J. Reinecke, Department of Nuclear Medicine, University Hospital Marburg, Marburg, Germany

Gerrit Ahlers, Department of Nuclear Medicine, University Hospital Marburg, Marburg, Germany

Andreas Burchert, Department of Internal Medicine, Hematology, Oncology and Immunology, University Hospital Marburg, Marburg, Germany

Friederike Eilsberger, Department of Nuclear Medicine, University Hospital Marburg, Marburg, Germany

Glenn D. Flux, Department of Physics, Royal Marsden Hospital and Institute of Cancer Research, Sutton, United Kingdom

Robert J. Marlowe, Spencer-Fontayne Corporation, Jersey City, NJ 07304-1901, USA

Hans-Helge Mueller, Institute for Medical Bioinformatics and Biostatistics, Philipps University of Marburg, Marburg, Germany

Christoph Reiners, Department of Nuclear Medicine, University Hospital Wuerzburg, Wuerzburg, Germany

Fenja Rohde, Department of Nuclear Medicine, University Hospital Marburg, Marburg, Germany

Hanneke M. van Santen, Department of Pediatrics, Wilhelmina Children's Hospital, University Medical Center Utrecht, Utrecht, The Netherlands; Princess Máxima Center for Pediatric Oncology, Utrecht, The Netherlands

Markus Luster (Corresponding Author), Department of Nuclear Medicine, University Hospital Marburg, Marburg, Germany

luster@med.uni-marburg.de
